# Evaluation of self-efficacy in Spanish nurse prescribers: are they qualified to safely and effectively prescribe medication and health devices?

**DOI:** 10.3389/fmed.2025.1663422

**Published:** 2025-11-26

**Authors:** Francisco Javier Gomis-Jimeno, Mojtaba Vaismoradi, Manuel Lillo-Crespo

**Affiliations:** 1 Department of Nursing, Faculty of Health Sciences, University of Alicante, Alicante, Spain; 2 HLA Vistahermosa Hospital, Alicante, Spain; 3 Faculty of Nursing and Health Sciences, Nord University, Bodø, Norway; 4 Faculty of Science and Health, Charles Sturt University, Orange, NSW, Australia

**Keywords:** nursing, interprofessional collaboration, nurse prescriber, nurse prescribing of medication, patient safety

## Abstract

**Introduction:**

In Spain the Royal Decree 1302/2018 of October 22nd regulates nurse prescribing of medication and health products (NPMHP) with at least 1 year of professional experience as a nurse. It provides nurse prescribers (NPs) with the authority to prescribe medications and health products, throughout established protocols published in the Official Spanish State Bulletin (BOE). In line with this, assessing the self-efficacy of NPs is relevant in its beginnings and by using tools such as the standardized nurse prescribing self-efficacy scale (NP-SES) it is possible to accurately evaluate their knowledge on NPMHP. This research aimed at exploring the Spanish accredited NPs’ perceptions on NPMHP knowledge and the impact of these prescribers’ confidence on their professional practice.

**Methods:**

A cross-sectional study was performed with Spanish NPs invited to complete the online version of the NP-SES. The cross-sectional study collected qualitative and quantitative data from a convenience sample of 14 NPs accredited in the last years at a health organization in Spain, encompassing various profiles, in terms of clinical specializations, complementary training and years of experience, for NPMHP.

**Results:**

All respondents expressed a positive perception of the implementation of NPMHP in the healthcare system and recognized NPMHP usefulness in enhancing patient safety, increasing patient satisfaction and therapeutic adherence, by strengthening interprofessional collaboration, and improving the efficiency of healthcare delivery. Specific areas where additional support and training may enhance nurses’ confidence and competence in prescribing roles have been identified.

**Conclusion:**

There is a need for education in the national undergraduate and postgraduate training of nurses regarding NPMHP as well as an adequate guidance on prescribing responsibilities in practice. In addition, the NPMHP protocols should be developed regarding nurses’ roles in prescribing medication within the multidisciplinary healthcare team. Future research should include prescribing audits to validate self-efficacy scores in the context where NPMHP takes place.

## Introduction

Perceived self-efficacy corresponds to confidence in one’s own abilities to be able to achieve expected results. Historically, Bandura et al. (47) defined self-efficacy as “people evaluate how capable they are of planning and carrying out the actions needed to achieve specific tasks. The focus is rather on their belief in what they can accomplish using those skills” (1). Currently, the concept of self-efficacy shows an individual’s belief in his or her ability to perform a behavior successfully (2). In 2019, a group of Spanish researchers developed and psychometrically examined Nurse Prescribing Self-Efficacy Scale (NP-SES), producing a reliable and valid instrument that can be used to assess the self-efficacy of the Nurse Prescribers (NPs) (3).

For decades, Pharmaceutical Care (PC) has been implemented and regulated in different ways across the world—especially across Europe—without a unified consensus among countries on the roles and competencies of different healthcare professionals, particularly in relation to prescribing of medication and health products (NPMHP) (4). Cipolle et al. in 2012, defined PC as the responsible provision of pharmacological therapy with the aim of achieving definitive results that improve the quality of life of patients by different professionals (5). Regarding the role of nurses, they are regularly involved in several medication roles such as the provision of information and patient education, monitoring medication, medication administration and identification of adverse drug reactions in the multidisciplinary healthcare team (6). Due to the increasing demand for health care especially with relation to the aging of population and the increase of chronic diseases and cognitive impairment, as well as the growing physicians’ and nurses’ shortages, the situation has derived in a societal urgent call for enhancing care quality, and lower healthcare costs. Therefore, NPMHP has gained momentum in several countries as one core strategy to face these situations (7, 8). Countries such as the United States, Australia, the United Kingdom, Ireland, Canada, Finland, New Zealand, the Netherlands, Sweden, Norway, and Spain have provided the independent prescribing authority to nurses in different forms as non-physician prescribers, albeit with distinct legal restrictions and regulatory frameworks (9–11). These new forms of prescribing, distinct from the traditional medical prescribing, have been referred to in both scientific and gray literature as follows: Nurse Prescribing, Nurse Prescribing of Medication (NPM), Nurse Prescribing of Medication and Health Products (NPMHP), and Non-Medical Prescribing (NMP).

In Europe, different initiatives have appeared such as the NuPhaC European Network which aims at promoting Nurses’ role in PC and specifically boosting NPMHP. In fact, the European Commission have funded projects related with PC and NPMHP such as DeMoPhaC, EUPRON or EQUANU aimed at developing a Pan-European model of PC based on interprofessional collaboration and communication and promoting the pivotal role of nurses. These initiatives seek to establish a unified framework and promote strategies in countries like Spain, where NPMHP is being implemented legally since 2018 at different speeds within the different regions’ health systems. Additionally, those initiatives are grounded in scientific evidence and keep demonstrating the competency of nurse prescribers (NPs) to prescribe medications and health products safely and efficiently, independently and in collaboration with physicians, pharmacists and other allied health professionals across Europe, characterized by person-centeredness (12, 13).

In Spain, Royal Decree 1302/2018, dated October 22nd regulated the indication, use and authorization of dispensing medications, health products and medical devices for human use by nurses. Under current Spanish regulations, nurses are authorized to prescribe certain medicines and medical products, including topical wound and burn treatments, ostomy care supplies, oral anticoagulation therapies, chronic disease management items, approved over-the-counter medications, and specified medical devices. NPMHP has been ruled by this decree since then and depends on a series of protocols and clinical guidelines published in the Official State Bulletin (BOE) (14) that each region is free to implement whenever they decide and is also free to regulate in terms of nurses’ profiles.

Currently, each of the Spanish 17 regions as autonomous communities are applying the Royal Decree differently in relation with the requirements for the nurses’ curricula to become accredited to prescribe under the protocols developed and approved by the Ministry of Health and published in the BOE. To date, the implemented protocols cover a range of conditions, including burns, wounds, diabetes mellitus, arterial hypertension, oral anticoagulation, ostomies, fever, smoking cessation, urinary tract infection, and the use of local anesthetics (15–24). However, the number of protocols functioning in each region nowadays is different as it depends on the regional government decisions.

Historically in Spain since 2008, nurse prescribing has been recognized as a role centered on caregiving and toward improving the quality of care. In its early stages, nurses were limited to prescribe care-related measures. However, professional discussions, debates and conflicts appeared later when prescribing was referred in 2018 as to NPMHP (25) and even though evidence shows that the implementation of NPMHP is linked to improved quality of care for patients, offering greater efficiency in terms of time, resource optimization and enhancing collaboration among healthcare professionals (26).

The Autonomous Community of Catalonia as a pioneer in accrediting for NPs in Spain has integrated the NPMHP to enhance primary care services more than other regions to date. NPs in this region manage a dedicated computer system that includes their personal identification card, enabling them to prescribe medications and healthcare products listed in regional guidelines. Since the introduction of this system, nearly 30,000 of Catalonia’s 52,000 active nurses have been accredited, prescribing around 172,000 treatments (27). However, as more NPs are accredited each day since the Royal Decree has been approved in Spain in 2018, the question that has emerged is to what extent are Spanish NPs truly qualified to safely and effectively prescribe medications, medical products and devices according to their own perception, or in other words what is their self-evaluation on the efficacy of what they are allowed to prescribe.

Despite significant strides in Europe to establish NPMHP as a vital component of PC, there remains a limited understanding of how this role is evolving within the complex and decentralized Spanish healthcare system. Although early evidence suggests that NPMHP enhances care quality, efficiency, and interprofessional collaboration, particularly in pioneering regions like Catalonia, there is a critical gap in evaluating whether newly accredited NPs are adequately prepared to prescribe safely and effectively. This study addresses this gap by exploring the competence and preparedness of NPs accredited within the last few years and according to their own perception and experience, thereby contributing with essential insights to inform policy, standardize training, and support the broader integration of NPMHP within Spain’s evolving primary care landscape.

## Objective

The aim of this study was to explore the accredited NPs’ perceptions on NPMHP knowledge and the impact of these prescribers’ confidence on their professional practice.

## Methods

### Design

A cross-sectional study was conducted through which a group of NPs were invited to complete a structured online questionnaire focused on self-efficacy including quantitative and qualitative data. The research report has been presented based on the STROBE Statement (Supplementary File).

### Sample and setting

The study population consisted of active NPs working in one of the three hospitals selected nationally by the Spanish Council of Nurses (SCN) for the pilot implementation of the NPMHP in private healthcare settings in Spain. This hospital was a private institution settled in Alicante and recognized for its participation in Nursing research projects and collaboration with the Board of Nurses in Alicante and the University of Alicante. To capture a range of professional perspectives within the hospital setting, participants were actively working in different clinical areas, including Medical/Surgical Hospitalization, Intensive Care/Hemodynamic/Critical Care Units, Emergency Services, Day Hospital, Primary/Community/Home Care, and Clinical Health Management/Teaching.

### Recruitment

A convenient sampling method with a maximum variation in the participants’ profiles in terms of NPMHP was used. They were definitely the total number of the nurses (*n* = 14) at the chosen Hospital and were all accredited to prescribe legally. This was the main reason why they were selected for the SCN piloting on the private receipt. This supported the depth and richness of information over breadth and rather tried to explore the lived experiences and perceptions of NPs regarding the implementation of NPMHP in their specific context. The inclusion criteria of the participants were nurses with at least 1 year of experience since the received their university undergraduate degree, that obviously had the official accreditation to prescribe. All the potential ones (14) agreed freely to participate once they received the invitation.

The principal investigators were recognized as expert nurses in NPMHP in the region and were the ones directly sending the invitations. In terms of academic background, the 14 NP participants were characterized by their additional training in NPMHP and had previously undertaken additional courses and modules on NPMHP organized by the University of Alicante and the Board of Nurses in Alicante, apart from their experience in practice that had qualified them as NPs according to the Spanish norms.

An email was sent to the 14 study participants in June 2024 containing an invitation to participate in the study, explaining the study objectives, informed consent form, and sociodemographic questionnaire. Soon after giving consent to participate in the study, they received an email containing a Google Forms link that granted direct access to the questionnaire, and another email in July 2024 as a follow-up reminder. Data collection took place between June and August 2024.

### Data collection tool

The Spanish version of the NP-SES questionnaire was used for the data collection (3) once having received the author’s permission for our study purpose. The content validity and reliability of the instrument was assessed before use. For reliability, its internal consistency and temporal stability were investigated. Its validity was assessed via exploring its content, criterion, and content validity. The criterion validity of the NP-SES was explored by correlating participants’ scores on the NP-SES and the Clinical Pharmacology Assessment Tool (CPAT). The NP-SES showed good internal consistency (Cronbach’s alpha = 0.958) and temporal stability (intraclass correlation coefficient = 0.783). Two dimensions of the CPAT as nursing care during drug administration and nursing care after drug administration were considered benchmarks because they assess the frequency with which nurses report performing tasks related to effective NPMHP.

The first section of the questionnaire (questions 1–12) gathered sociodemographic information from the participants such as gender, age, current professional practice, academic level, work area, geographic location, years of experience, accreditation as a NP, training in medication prescription, number of training hours, and the institution where this training was received. The second section (questions 13–31) comprised the validated Spanish version of the NP-SES questionnaire, designed to assess self-efficacy among NPs for independent prescribing specifically evaluating quantitatively and qualitatively the level of self-efficacy in NPMHP.

Responses were recorded using a drop-down scale ranging from 0 to 100 in 5-point increments, where 0 indicated “Totally sure that I cannot do it,” 50 represented “Moderately sure that I can do it,” and 100 signified “Totally sure that I can do it.” The NP-SES scores have been categorized into the following ranges: extremely low (0–28 points), very low (29–43 points), low (44–58 points), moderate (59–74 points), high (75–89 points), and very high (90–100 points). This format was used to facilitate consistent responses and minimize data entry errors. In addition, at the end of the questionnaire and with aim of highlighting the participants’ experience, they were invited to openly provide free comments on the questionnaire items (3). The estimated time for the completion of the NP-SES questionnaire was approximately 10 min.

### Ethical considerations

This project was approved by the Research Ethics Committee of the Faculty of Health Sciences at the University of Alicante. Participant confidentiality was safeguarded in accordance with the European General Data Protection Regulation (GDPR), Regulation (EU) 2016/679, and Spain’s Organic Law 3/2018 of December 5, on the Protection of Personal Data and Guarantee of Digital Rights.

Informed consent was obtained from all participants prior to their involvement in the study. To ensure anonymity, each participant was assigned an alphanumeric code, with identities known only to the research team. All collected data are securely stored on an encrypted external hard drive maintained by the principal investigator.

### Data analysis

Descriptive statistics were used for data analysis. Measures such as the mean, standard deviation, mode, and median were calculated, with a focus on frequencies and percentages. The primary objective was to assess the level of self-efficacy among NPs across various hospital units. The data analysis was primarily quantitative, except for the final section of the questionnaire, in which the qualitative data of the study were collected using the Google Forms Statistics tool, through a standardized questionnaire developed by the researchers.

The free-text responses at the end of the questionnaire were subjected to content analysis by the first author, with supervision from a team member to enhance analytical rigor and ensure credibility and dependability. Accordingly, patterns and meanings in the qualitative data were identified. Responses were first read thoroughly to gain an overall understanding, followed by open coding to label key concepts and recurrent ideas. These codes were then grouped through iterative comparison and refinement. The analysis was conducted manually, ensuring consistency and consensus in the coding process. Representative quotations from participants were selected to support and illustrate the interpretation of findings (28).

## Results

### Sociodemographic characteristics of the participants

The study participants were predominantly women (80%, *n* = 11), with an average age of 39 years. The remaining 20% were men (*n* = 3), with an average age of 36 years. In terms of education level, they were diverse as seven women held a nursing degree, one woman had a postgraduate degree, three men held a master’s degree, and one had doctoral degree. Regarding work experience, one group (*n* = 6) consisted of three women and three men had less than 10 years of experience, and another group (*n* = 8) consisting of seven women and one man had 20–30 years of experience.

The study participants were working in various units at the Hospital Center. The group (*n* = 8) including seven women and one man worked in hospitalization, one woman in the day hospital, two women in primary care, one man in management/teaching, and one woman and one man in the intensive care unit.

Regarding accreditation as NP, all participants in the study were accredited, except for one man who worked as a manager and teacher, but she was recruited given her active involvement in the pilot project and being in the final stage of the accreditation process. Among the female participants, nine had specific training in NPMHP, while four women had little to no specific training despite being accredited. They had extensive national and international publications on nurse prescribing, whose insights were valuable to the research.

Notably, three participants completed specifically accredited NPMHP training at the two reference centers in Alicante. The number of hours of accredited specific training in NPMHP was as follows: three women and one man did not have any specific NPMHP training; four women with up to 10 h of specific training; two women with up to 20 h of training; and two men and one woman with over 20 h of specific training.

They were asked about their preferences regarding the Clinical Practice Guidelines published in the BOE. The distribution of preferences was as follows: wounds (*n* = 6), burns (*n* = 6), ostomies (*n* = 3), oral anticoagulants (*n* = 3), diabetes mellitus (*n* = 4), local anesthetics (*n* = 2), fever (*n* = 1), and antihypertensives (*n* = 1) (Table 1).

**Table 1:** Sociodemographic characteristics of participants (*n* = 14).

Variable	*n* (%)
Hospital	
HLA Vistahermosa	14 (100)
Gender	
Female	11 (80)
Male	3 (20)
Age	
20–30	3(21.45)
31–40	3 (21.45)
41–50	4 (28.60)
+50	3 (21.45)
Academic background	
Undergraduate	7 (50.05)
Postgraduate	4 (28.6)
Doctorate	3 (21.45)
Years of work experience	
<10	6 (42.8)
10–20	0 (0)
20–30	8 (57.2)
Do you currently work as a nurse?	
Yes	13(92.85)
No	1 (7.15)
Clinical practice area	
Hospitalization	8 (57.2)
Day hospital	1 (7.15)
Primary care	2 (14.3)
Management/teaching	1 (7.15)
Intensive care unit	2 (14.3)
NPMHP accreditation	
Yes	13 (92.85)
No	1 (7.15)
NPMHP specific training	
Yes	9 (64.3)
No	5 (35.7)
NPMHP training center	
University of Alicante	1 (11.1)
College of Nursing of Alicante	5 (55.5)
Both	3 (33.1)
NPMHP training hours	
0	5 (35.05)
< 10	4 (28.9)
10–20	2 (14.3)
> 20	3 (21.6)
NPMHP clinical practice guidelines (BOE)	
Wounds	6
Burns	6
Ostomies	3
Oral anticoagulation	3
DM	4
Local anesthetics	2
Fever	1
HTA	2

NPMHP, Nurse Prescribing of Medications and Health Products; DM, Diabetes Mellitus; HTA, Antihypertensives; BOE, Official State Gazette.

### Spanish nurses’ self-efficacy in NPMHP

The analysis of participants’ responses revealed varying levels of self-efficacy across the three assessed domains. In the area of *Clinical Evaluation and Pharmacological Knowledge* (items 1–5), nurses reported low self-efficacy, with a mean score of 49.00 (SD = 19.48), indicating a need for further training in pharmacological assessment and decision-making. In contrast, self-efficacy was high in *Complementary Prescribing and Evidence-Based Practice* (items 6–10), with a mean score of 77.14 (SD = 14.18), suggesting greater confidence in integrating evidence-based guidelines and complementary approaches into prescribing decisions. Lastly, *Independent Prescribing and Patient Education* (items 11–19) also showed a low self-efficacy score of 57.77 (SD = 17.77), pointing to potential challenges in independent prescribing and delivering patient-centered education. The total NP-SES mean score was 60.56, reflecting a moderate level of self-efficacy in NPM among the NPs.

These findings highlight specific areas where additional support and training may enhance nurses’ confidence and competence in prescribing roles (see Table 2).

**Table 2:** Self-efficacy in NPM among the NPs using the NP-SES questionnaire.

Item	Mean	SD*	Mode	Median
Clinical evaluation and pharmacological knowledge				
Item 13	55	21.01	50	80
Item 14	55	15.04	50	60
Item 15	45	21.90	100	85
Item 16	40	19.94	80	75
Item 17	50	19.49	50	80
Complementary prescribing and evidence-based practice				
Item 18	45	20.75	100	80
Item 19	86,42	10.08	100	100
Item 20	89,28	14.39	100	95
Item 21	95	12.77	100	90
Item 22	70	12.92	100	95
Independent prescribing and patient education				
Item 23	55	22.67	100	85
Item 24	45	19.79	50	70
Item 25	45	20.82	80	75
Item 26	45	20.13	80	80
Item 27	50	19.49	50	70
Item 28	45	19.91	100	95
Item 29	90	11.67	100	95
Item 30	70	14.52	70	90
Item 31	75	10.89	80	80

*SD, Standard Deviation. Scale scoring: extremely low (0–28 points), very low (29–43 points), low (44–58 points), moderate (59–74 points), high (75–89 points), and very high (90–100 points).

The participants’ qualitative comments indicated that they unanimously agreed on the usefulness of the questionnaire in identifying gaps in their knowledge as NPs. Additionally, they highlighted the scarcity of information available to nursing professionals on the topic and suggested the possibility of incorporating and expanding university-level training in NPMHP.

Some of the most relevant comments made by the participants were referred to the need of increasing the number of medications and health products that NPs might prescribe in Spain, as well as improving NPMHP training at higher education institutions and enhancing the competencies and conditions of nurse prescribers:

*“…at the moment in Spain there are many different types of guidelines or protocols for different chronic pathologies and situations, but we need to increase the number of these guidelines to cover a larger number of the population’s situations…”* (Participant 4)

“…*NPMHP training should be introduced at universities, within undergraduate nursing education levels and also provided as postgraduate or master's degree levels; it is the only way to encourage nurses to learn reliable and up-to-date knowledge…we have learned about it in practice…however I see new students arriving every day to our organization with no idea about NPMHP…*” (Participant 7)

*“…Current clinical practice guidelines in NPMHP should focus more on the role, the working conditions, the workplace loads of the nurse prescriber…especially the governments should promote more these new competencies…it is ok to be able to prescribe however our salary is the same though our workload and responsibilities is increasing…”* (Participant 1)

“…*the only way to be socially recognized by the population is to invest in quality university education in NPMHP*…” (Participant 13)

## Discussion

This cross-sectional study aimed to explore the professionals’ perceptions of NPs regarding prescribing knowledge and the impact of NPs’ confidence on their professional practice. Accordingly, all respondents viewed NPMHP positively, recognizing its benefits in improving care quality, patient satisfaction, therapeutic adherence, interprofessional collaboration, and healthcare efficiency. Within the multidisciplinary healthcare team involved in medication management, NPs play a crucial role in medication management as their practices improve medication safety and enhance the quality of life of patients, easing the burden on healthcare systems and caregivers (29). Regarding the NP-SES, it is a psychometrically sound instrument in the Spanish context and can be used to evaluate nurses’ self-efficacy in nurse prescribing (3). The analysis of participants’ responses revealed varying levels of self-efficacy across the three assessed domains. NPs require to be well-qualified to prescribe medications and medical devices following BOE protocols. In the areas of *Clinical Evaluation and Pharmacological Knowledge and Independent Prescribing and Patient Education*, the NPs reported low self-efficacy. They highlighted potential challenges in autonomous prescribing and delivering patient-centered education. This aligns with the participants’ comments indicating a need for further training in pharmacological assessment and decision-making. In contrast, self-efficacy among NPs was high in *Complementary Prescribing and Evidence-Based Practice* suggesting greater confidence in integrating evidence-based guidelines and complementary approaches into prescribing decisions. The current evidence suggests that low self-efficacy may hinder NPs’ ability to prescribe medications effectively (30, 31), which in turn plays a crucial role in the development of their competencies as NPs (32). Additionally, the results of a pioneering study in Spain on self-efficacy in NPs have shown several blocks related to NPMHP, including pharmacological knowledge, clinical assessment, complementary prescribing, evidence-based practice, independent prescribing, and patient education (3).

NPs felt positive about NPMHP despite low confidence in pharmacology because they recognize its potential to enhance patient care, improve workflow efficiency, and strengthen professional autonomy. NPs value the ability to respond more quickly to patient needs, reduce delays in treatment, and increase continuity of care—benefits that often outweigh their initial uncertainty in pharmacological knowledge (33, 34). NPMHP promotes professional development and can contribute to the redesign of primary care, making it more sustainable while enhancing the self-efficacy of NPs (35, 36). Although the existence of different legal frameworks for NPM around the world has led to varying organizational models, the high self-efficacy of NPs remains evident, despite the complexities in implementing NPM within health systems. In Spain, particularly in Catalonia, NPs believe that the current drug catalog available for prescription is insufficient for autonomous prescribing. They are advocating for the expansion of this catalog to enhance patient care and improve quality (37).

NPs demonstrated moderate self-efficacy in the domain of Independent Prescribing reflecting a cautious level of confidence. Some participants mentioned that even when NPs felt capable, this confidence might not be fully expressed in practice without appropriate structural and organizational support. The effectiveness of nurse prescribing is highly dependent on system-level factors such as clear role definitions, supportive infrastructure, and manageable workloads (38).

While progress in professional accreditation in Spain and regulatory changes in Catalonia is acknowledged, there are no conclusive studies that thoroughly evaluate the safety of NPMHP and its impact on patient satisfaction in this context (39). Currently, Spanish NPs have access to a set of protocols that enhance their self-efficacy, enabling them to make clinical decisions once solely made by physicians. Existing literature on the substitution of physicians by NPs indicates that NPs perform equally well, without an increase in adverse events (40). Internationally, studies in the UK and US have shown a significant rise in NPM for patients with chronic diseases and the prescription of medical devices. They highlight the economic benefits, improved patient satisfaction, and enhanced treatment adherence as key advantages of NPM (41–43).

Finally, NPs’ commitment to promoting and encouraging accreditation as prescribers involves the adoption of health policies and nurse management support to strengthen NPMHP in Spain. This includes the development of essential resources, such as professional training and the provision of necessary materials, to ensure the correct implementation of NPMHP in the healthcare system, ultimately ensuring quality care. Robust models of NPM are being considered globally to address population needs. The results can contribute to the future implementation of independent non-physician prescribing and guide government and society on interventions that can be used to consolidate it. The shortcomings of traditional physician-led systems of care mean that new approaches are imperative to maintain patient access to prescription drugs, with NPs being a key element in this regard (44–46).

## Limitations

One limitation of this study is that the sample was drawn from a single hospital, which may limit the generalizability of the findings to other settings. Even though the study was carried out in just one hospital it is important to point out that was one of the three selected nationally by the SCN for the piloting of NPMHP private receipt and all the nurses participating in such pilots accepted to be part of our study and provided their experience in different clinical areas. While this setting allowed for an in-depth exploration of early experiences with NPMHP implementation especially as the qualitative perspective was pertinently included. The insights gained from this setting are still valuable, as they highlight challenges and facilitators of NPMHP in a less-studied sector of the healthcare system such as the private one. Future studies should aim to include a broader range of settings, particularly public hospitals and other private ones in other regions, to provide a more comprehensive understanding of NPMHP implementation across Spain.

In addition, the NP-SES reflects perceived, not actual competence, introducing self-report bias. Also, this study focused on a specific group of participants from a particular geographic area in Spain, which may not reflect the experiences or perspectives of NPs in other regions or countries. Expanding the study to include a broader geographic scope, or even an international perspective can offer more comprehensive insights into NPMHP and self-efficacy. Future studies should include NPMHP audits to validate self-efficacy scores in real practice.

## Conclusion

This study’s focus on self-efficacy among NPs in Spain highlights a timely and evolving challenge as nurses strive to integrate prescribing competencies into their professional roles. The implementation of NPMHP was viewed positively in this research as NPs believed that NPMHP enhanced the quality of care, improved patient satisfaction and therapeutic compliance, and streamlined healthcare delivery. This study also indicated a gap in national training for NPMHP as many nurses lack comprehensive knowledge about their prescribing responsibilities and the effectiveness of prescribing certain medications based on clinical practice guidelines or protocols published by the Ministry of Health. There is a need to address training gaps and increase the number of education hours in pharmacology, clinical assessment, and evidence-based prescribing to ensure that NPs are fully prepared to prescribe medications and medical products safely and effectively. Enhancing formal education in these areas is essential to build confidence, ensure competence, and align with the legal and clinical responsibilities required for independent prescribing.

As Experts’ voices such as NuPhaC Network advice, NPMHP in Spain, as in other European contexts, should be grounded in a multidisciplinary PC framework with the participation of nurses, physicians and pharmacists that prioritizes patient safety while also fosters self-confidence among the professionals responsible for NPMHP. Therefore, it is essential to prioritize protocols for chronic diseases (e.g., COPD, mental health) where NPs’ role is underutilized and to pilot autonomous prescribing along with competency assessments to evaluate safety.

## Data Availability

The original contributions presented in the study are included in the article/Supplementary material, further inquiries can be directed to the corresponding author.
